# Synergistic Efficacy of Plaque Control with Intralesional Triamcinolone Acetonide Injection on Erosive Non-Gingival Oral Lichen Planus: A Randomized Controlled Clinical Trial

**DOI:** 10.3390/ijerph192113787

**Published:** 2022-10-23

**Authors:** Wei Zhao, Duanxian Lin, Shuzhi Deng, Shimeng Wang, Yiqing Guo, Jin Yang, Xueke Shi, Hongmei Zhou

**Affiliations:** 1 State Key Laboratory of Oral Diseases, National Center of Stomatology, National Clinical Research Center for Oral Diseases, Frontier Innovation Center for Dental Medicine Plus, West China Hospital of Stomatology, Sichuan University, Chengdu 610041, China; 2 Department of Oral Medicine, Qingdao Stomatological Hospital, Qingdao 266001, China

**Keywords:** oral lichen planus, erosive non-gingival lesions, plaque control, intralesional injection, triamcinolone acetonide

## Abstract

This study is the first time to assess the synergistic efficacy and safety of plaque control on erosive non-gingival oral lichen planus (OLP). A randomized, controlled, clinical trial with blind evaluation was designed, and 48 OLP patients with erosive non-gingival OLP lesions were randomly assigned to the experimental group (*n* = 25, receiving intralesional triamcinolone acetonide injection, periodontal scaling, and oral hygiene instruction) and the control group (*n* = 23, only receiving intralesional triamcinolone acetonide injection) once a week for 2 weeks. Erosion size, pain level, plaque index, and community periodontal index were measured at every visit. Patients cured of erosion were followed up for 3 months to evaluate the recurrence rate. Adverse reactions were also recorded. At day 14 ± 2, the experimental group showed a higher completely healed percentage of erosion, a greater reduction of erosion size and pain level. However, no significant difference was observed in the recurrence rate. No participants had any severe adverse reactions. In conclusion, an improvement was observed in patients with plaque control, and future studies with larger sample sizes are needed to reinforce the external validity of this study.

## 1. Introduction

Oral lichen planus (OLP) is a chronic inflammatory mucocutaneous disease with a global prevalence of 1.01% [1]. OLP involved most frequently the non-keratinizing buccal and tongue membrane [1]. The two main clinical forms of OLP are white and erosive lesions. White lesions are mostly asymptomatic, while erosive lesions can result in pain and intense discomfort [1]. Some patients have a high incidence of relapse [2,3,4]. Therefore, erosive OLP patients suffer from reduced quality of life. To be noted, recent studies indicated that the malignant transformation rate of OLP was between 0.44% and 2.28% [5,6,7,8,9]. The erosion is a risk factor for OLP malignant transformation [6,9], and the malignant transformation rate of lesions in the tongue and the buccal membrane is much higher than that in the gingiva [5,6,7,9]. Hence, controlling inflammation effectively to accelerate the relief of symptoms and remission of erosive lesions is the primary target for erosive OLP.

At present, topical corticosteroids are generally preferred for erosive OLP resulting from the significant anti-inflammatory effect [10,11,12,13,14,15]. Several corticosteroids have been widely used in the topical treatment of OLP, including triamcinolone acetonide (TA), clobetasol propionate, betamethasone sodium phosphate, dexamethasone, clobetasol, fluocinonide, and fluticasone propionate [10,11,12,13,14,15]. TA is a commonly used corticosteroid drug, and previous studies demonstrated that clobetasol propionate is a high-potency topical corticosteroid that is used as a primary alternative drug to TA [16,17]. Usually, a corticosteroid rinse or paste is applied and when an erosive spot does not heal well or always recurs, one will consider an intralesional injection [12,13]. However, there are still some patients who are refractory to topical corticosteroids. Therefore, it is imperative to find other ways to improve the efficacy of topical corticosteroids.

The periodontal inflammation, as a source of local irritation, is identified as a factor exacerbating gingival lesions of OLP [18,19,20,21,22]. Active management of oral hygiene contributes to alleviating gingival OLP lesions [18,19,20,21,22]. Non-gingival lesions in the buccal or ventral tongue membrane are more frequent and may also be affected by periodontal inflammation. However, the relationship between erosive non-gingival lesions and periodontal inflammation has not been studied yet. Clinicians are also uncertain if dental plaque control strategies including periodontal scaling and tooth brushing could mechanically irritate or relieve the erosive non-gingival lesions.

Therefore, we designed this randomized controlled prospective trial with blind evaluation to study the synergistic effect of plaque control on erosive non-gingival OLP. A total of 48 patients with erosive OLP were enrolled and randomly divided into two groups with an allocation ratio of 1:1. Patients in the experimental group underwent TA injection, periodontal scaling, and oral hygiene instruction, the patients in the control group only received TA injection. Procedures in both groups were conducted once a week for 2 weeks. The completely healed percentage, OLP erosion size, and pain level in the two groups were compared at day 14 ± 2, and the recurrence rate was recorded after 3 months. Overall, our study aims to evaluate the short-term synergistic efficacy and safety of plaque control in the treatment of erosive non-gingival OLP.

## 2. Materials and Methods

### 2.1. Participants

From May 2013 to January 2014, eligible OLP patients with non-gingival erosions were enrolled at the Department of Oral Medicine, West China Hospital of Stomatology, Sichuan University. All patients were diagnosed with OLP according to the World Health Organization criteria earlier for more than 2 months [23]. The study protocol was approved by the Ethics Committee of West China Hospital of Stomatology, Sichuan University (WCHSIEC-CR-2012-0001) and registered at http://clinicaltrials.gov/, accessed on 16 October 2022 with the ID (ChiCTR-TRC-13004128). Written informed consent to participate in the research was obtained from all participants. The study procedures were performed in accordance with the tenets of the Declaration of Helsinki, and relevant local laws and regulations.

The inclusion criteria were as follows: (1) age: 20 to 60 years old; (2) OLP duration: more than 2 months; (3) the erosive lesion located in the buccal or tongue membrane that was close to gingiva, and manifested a central area of shallow ulceration; (4) the erosive lesion in that area was frequently relapsed or did not heal completely after 2-week corticosteroid rinse or paste; (5) there was one single erosion of less than 100 mm^2^; (6) patients with chronic gingivitis or mild chronic periodontitis (1 ≤ plaque index (PI) ≤ 5 and 1 ≤ community periodontal index (CPI) ≤ 3) [24,25], and receiving no periodontal scaling within the previous 6 months; (7) normal physical examination results before intervention (including blood pressure, blood routine, blood glucose level, hepatic and renal clinical biochemistry, routine urinalysis, ultrasonic examination of abdomen, X-ray of chest and electrocardiogram).

Patients with the following conditions were excluded: (1) patients with other severe oral mucous diseases; (2) patients with a history of immunomodulating drug treatment in the preceding 3 months or topical medication of OLP lesions within the previous 1 week; (3) pregnant or lactating women; (4) patients with poor compliance; (5) patients with erosions caused by trauma or chemical irritation.

All researchers and clinicians accepted professional training in the diagnostic criteria and research procedures. Basic information about the patient’s age, sex, OLP duration, medical history, medication history, family history, clinical manifestations, and periodontal status were recorded at the first visit.

### 2.2. Randomization

This design was a randomized controlled prospective trial with blind evaluation. Each patient enrolled in the study was allocated a random number by a computer-generated random number list. Those with odd numbers were allocated to the experimental group, and those with even numbers were assigned to the control group. The allocation ratio is 1:1.

### 2.3. Study Interventions

The patients in the experimental group were treated with intralesional TA injection, periodontal scaling, and oral hygiene instruction, while the patients in the control group were treated with intralesional TA injection alone.

The intralesional injection was performed as follows: a TA injection (1 mL, 40 mg, Laboratorio Italiano Biochimico Farmaceutico Lisa pharma SPA, Erba, Italy) was mixed with an equal volume of sterile water. A single-point injection (0.5 mL, 10 mg TA) was given into the connective tissue below the erosive lesion from the adjacent normal mucosa, once a week for 2 weeks. The patients were observed for 20 min after the injection, in case of any possible adverse reactions.

Periodontal scaling of the experimental group was practiced using ultrasonic scalers (Minipiezon, EMS, Nyon, Schwitzerland) under a standardized set of conditions: 3 × 6 mm area, ≤15–degree angle between the scaler tip, adaptation of the terminal 2–3 mm of the tip of the instrument to the tooth and 40–80 g of lateral pressure at the rate of 12 strokes/10 s [26]. The procedure was performed carefully and delicately, to avoid irritating the lesions. Supragingival scaling was performed in all patients in the experimental group, while subgingival scaling was carried out only when subgingival calculus was found. The tooth surfaces were polished after scaling with a rubber cup and polishing paste, using a micromotor at a controlled speed, thus avoiding injury to the adjacent lesion. Additionally, those treatments were carried out by a single, specially trained periodontist. Following completion of the treatment, only patients in the experimental group were offered the same soft-bristled toothbrushes and toothpaste, and they were expected to brush teeth 3 times a day after every meal throughout the study. For them, oral hygiene instruction was carried out. Control subjects continued with their routine dental plaque control regime without oral hygiene instruction.

In addition, it was recommended that the patients in both groups should avoid spicy food and other topical or oral medications throughout the study.

### 2.4. Clinical Assessment

The erosion size (mm^2^), pain level (numeric rating scale, NRS) [27], PI, and CPI were recorded at the first day and 14 ± 2 days later. Measurement of the maximum diameter (mm) and width (mm), perpendicular to each other, was made with a calibrated periodontal probe. The area of erosion (mm^2^) was calculated by multiplying the maximum diameter and the maximum width. The NRS was a horizontal line divided into 10 equal segments indicating no pain (0) to extreme pain (10) [27]. We explained to the patients how to use the NRS before measuring the pain level, which was performed by gently wiping the erosion with a sterile swab and then asking the patients to select a number from 0 to 10 representing their pain level. For the PI, the Turesky-modified Quigley–Hein plaque index was applied to evaluate the state of dental plaque adhesion (the third molar was excluded) [24]. CPI measurements were performed with a periodontal probe in 6 regions of the oral cavity to examine the gingival bleeding, dental tartar, and pocket depth, and the highest scores were recorded as the results [25].

### 2.5. Adverse Reactions

Possible adverse reactions in the two groups were recorded in detail during and after the oral examination and the intralesional injection. If there was any adverse event, the patient would be kept under observation. If the adverse event was serious, then the experiment would discontinue, and the subject would be referred to a physician for overall therapy.

### 2.6. Blind Evaluation

A blind evaluation was used in this trial. All patients and the researcher who carried out the treatment knew the therapeutic regimen. However, the researcher who carried out the treatment did not participate in data recording and analysis. A different investigator who took the measurement was not told the treatment details. Every evaluation was performed by another researcher who was blinded to the group allocation.

### 2.7. Follow-Up Assessment

The patients in the experimental group and control group whose erosion completely healed after one or two injections were followed up to detect the recurrence rate after 3 months. If the patients’ erosion did not heal completely after 2-week treatment, conventional therapy would be applied to accelerate the healing process. Patients in the control group who accomplished the trial were given the same plaque control measurements as the experimental group.

### 2.8. Statistical Analysis

**Sample-Size Calculation.** The sample size was calculated based on a previous pilot study conducted by our research group. The ratio of sample numbers in the two groups was 1:1, we considered the power (1 − beta) to be 80%, with a significance level (alpha) of 0.05; according to the pilot study, the completely healed percentage of the control group was around 51%, and the completely healed percentage of the experimental group was around 95%. We calculated the sample size by PASS 11.0 (NCSS LLC, Kaysville, UT, USA), and it should be a total of 40 subjects. Allowing for the 20% dropout rate, the number of samples required for inclusion in the study would be 48.

Statistical analysis was performed with SPSS 21.0 (Armonk, NY, USA: IBM Corp.). For continuous variables including erosion area, NRS, PI, and CPI, the mean ± standard deviation was used to represent the distribution of individual values of each group, and independent 2-sample *t*-test was used to analyze the differences. Moreover, effect sizes are calculated as a standardized mean difference by Cohen’s d methods for continuous outcomes, 95% confidence interval (CI) was also provided following Cohen’s d value showing as Cohen’s d (95% CI) [28]. We ranked the level of Cohen’s d effect size following the standard below: Cohen’s d < 0.2: the difference is negligible, 0.2 ≤ Cohen’s d < 0.5: the difference is small, 0.5 ≤ Cohen’s d < 0.8: the difference is medium, Cohen’s d ≥ 0.8: the difference is large [29]. While, for dichotomous outcomes, chi-squared test was used to analyze the *p* values, and the effect size was shown with odds ratio (95% CI). Effect sizes were only calculated for outcomes at day 14 ± 2 and 3-month visits, but not the variables at the baseline, i.e., first visit, when no treatment was applied, and no outcome was shown at that time. All statistical tests were performed using a significance level of *p* < 0.05.

## 3. Results

### 3.1. Participants

**Figure 1:**
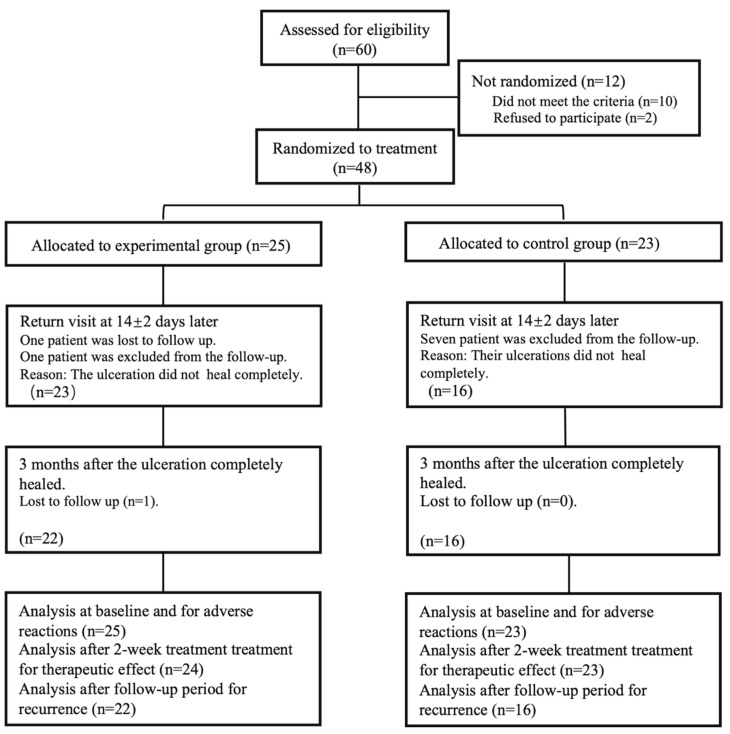
The flow diagram of the trial.

A total of 48 participants including 15 men (31.2%) and 33 women (68.8%) aged 29 to 60 years were enrolled in this trial, including 25 in the experimental group and 23 in the control group. One patient in the experimental group did not return to visit at day 14 ± 2, and one did not return at 3-month follow-up; therefore, 46 patients (23 patients in each group) completed the trial. The expulsion rate was 4.2% (2 of 48). The flow diagram of the trial is shown in Figure 1.

### 3.2. Baseline

Baseline demographic characteristics of age, sex, position, and OLP duration are shown as Table 1. The baseline was identical for both groups.

### 3.3. Efficacy Analysis

**Intergroup Comparison.** A higher completely healed percentage at day 14 ± 2 was found in the experimental group (95.8% vs. 69.6%, χ^2^ = 4.03, *p* = 0.045*). The odds ratio (95% CI) is 10.06 (1.13, 89.94), demonstrating that the healing rate in the experimental group is 10.06 times greater than the control group (Table 2).

At day 14 ± 2, greater reductions in erosion size and NRS score were found in the experimental group with medium Cohen’s d effect size: the reduced erosion area (19.1 ± 12.8 mm^2^ vs. 10.3 ± 13.2 mm^2^, *p* = 0.029*, effect size: 0.68 (0.08, 1.28)), and the reduced NRS level (3.9 ± 1.7 vs. 2.6 ± 2.4, *p* = 0.036*, effect size: 0.63 (0.03, 1.23)) (Table 3).

At day 14 ± 2, there was still one patient in the experimental group and seven patients in the control group whose erosion did not heal completely. All those eight patients were given oral glucocorticoid therapy (0.5 mg/kg/d, taken at a draught in the morning for 7 days) and 0.1% chlorhexidine rinsing the mouth (10 mL once, 3 times daily). Only one patient in the control group did not heal after the glucocorticoid therapy, then he received microwave treatment and the erosion healed completely.

### 3.4. Improvement of Periodontal Status

**Intergroup Comparison.** At day 14 ± 2, the greater reduction of PI and CPI were observed in the experimental group with a large Cohen’s d effect size: reduced PI (1.7 ± 0.9 vs. 0.6 ± 0.8, *p* < 0.001***, effect size: 1.29 (0.65, 1.93)), and reduce CPI (1.6 ± 0.9 vs. 0.5 ± 0.7, *p* < 0.001***, effect size: 1.36 (0.71, 2.01)) (Table 3).

### 3.5. Recurrence Analysis

A total of 39 patients (23 in the experimental group and 16 in the control group) showed complete healed erosion at day 14 ± 2 and were placed into the follow-up group to assess the recurrence rate 3 months later. The results of 3-month follow-up showed that there was no significant difference in recurrence rate between the experimental group (7/22, 31.8%) and the control group (6/16, 37.5%) (χ^2^ = 0.13, *p* = 0.715, odds ratio 1.29 (0.33, 4.97)) (Table 4).

### 3.6. Safety Analysis

None of participants suffered any serious adverse events. Only one participant in the experimental group had a minor adverse reaction, which manifested as mild dry mouth 4 h after the intralesional injection. The symptoms disappeared spontaneously after 1 h without treatment.

## 4. Discussion

To our knowledge, this is the first randomized controlled clinical trial to evaluate the synergistic efficacy of plaque control with local corticosteroid on erosive non-gingival OLP. Since our patients’ erosions frequently relapsed in the same area or did not heal completely after a 2-week corticosteroid rinse or paste, we chose intralesional injection as the medication. Intralesional TA injection has been known to be a successful treatment for erosive OLP with fewer adverse reactions in our or other’s several studies [4,30,31]. Furthermore, since intralesional TA injection was performed by one professional doctor instead of the patients themselves, medication application in the two groups was identical. Therefore, we selected intralesional TA injection as the medication for this study. In the phase of the pilot study, another experimental group, treated with periodontal scaling and oral hygiene instruction without TA injection, was rejected by the ethics committee because it cannot make the erosive non-gingival lesions of OLP heal completely.

This present study demonstrated that the experimental group experienced a higher completely healed percentage, a more considerable reduction of erosion size or pain score, and a greater decrease in PI and CPI than that in the control group. Consequently, plaque control has a synergistic effect with medication on erosive non-gingival OLP. Holmstrup et al. [20] and Salgado et al. [21] both found that plaque control consisting of supragingival scaling and oral hygiene instruction was effective in improving the clinical features and painful symptoms of oral lichen planus with gingival involvement. More recently, a randomized controlled study also demonstrated that a structured plaque control intervention was effective in improving the gingival OLP lesions [32]. These studies merely evaluated the effect of periodontal treatment with no concomitant medication on the improvement of gingival lesions in OLP. However, it is not common to treat OLP merely by plaque control. Topical corticosteroid is generally preferred for the treatment of OLP. Therefore, it is much more worthwhile to study the synergistic effect of plaque control with medication on OLP.

López-Jornet et al. [18] and Guiglia et al. [33] evaluated the efficacy of plaque control and administration of a topical corticosteroid, respectively. These two studies confirmed that effective plaque control had an adjuvant effect with concomitant medication on gingival lesions of OLP. Our study was somewhat similar to the two studies. However, they are all pre- and post-test descriptive clinical studies with no control group. In addition, they did not evaluate the more frequently involved non-gingival lesions. Our study firstly demonstrates that plaque control exerted a positive effect concomitant to medication treatment on the improvement of erosive non-gingival lesions in OLP. Therefore, the results of our study will provide evidence to dentists that periodontal scaling can be conducted when OLP patients have erosive non-gingival lesions.

**Figure 2:**
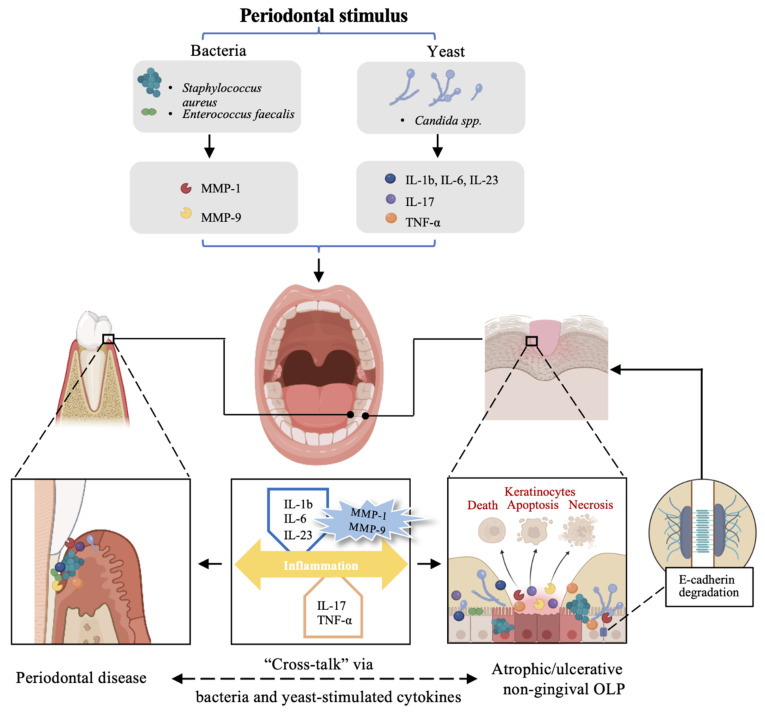
The hypothesis of “cross-talk” between periodontal and mucosal inflammation in OLP patients with non-gingival lesions. On one hand, bacteria and yeast-associated dental calculus or plaque could promote E-cadherin degradation, oral keratinocyte cell death, apoptosis, and necrosis, aggravating mucosal damage. On the other hand, cytokines stimulated by bacteria (MMP-1, MMP-9) and yeast (IL-1b, IL-6, IL-13, IL-17, TNF-α) could accelerate inflammation in periodontal tissue and mucosal tissue. Removement of dental calculus and plaque could reduce yeast, bacteria, and inflammatory cytokines, subsequently, contributing to the healing of mucosal erosion and the alleviation of periodontitis. Meanwhile, the healing of OLP erosion also reduced cytokines that promoted the development of periodontitis, with an improvement of the periodontal condition.

An interesting phenomenon captured our attention, in that the PI and CPI witnessed a decrease in both groups. It is reasonable that periodontal scaling and daily personal plaque control improved the periodontal status with decreased PI and CPI in patients of the experimental group. What resulted in the decreases in PI and CPI in the control group? We inferred that healed erosion and reduced pain allowed adequate self-performed plaque control, leading to a better periodontal status. Moreover, we inferred the bilateral interaction between periodontal disease and OLP, and molecular mechanisms also support our hypothesis. Matrix metalloproteinase (MMP)-1 and MMP-9 which played an important role in both diseases are upregulated in OLP patients with periodontal diseases [34]. In a longitudinal study, Romano et al. [35] provided evidence that bacterial plaque stimulates MMP secretion and may contribute to extracellular matrix degradation of oral mucosa and gingiva. Consequently, we tentatively put forward that the improvement of periodontal status or mucosal condition may decrease the inflammation cytokines including MMP-1 and MMP-9, and then inversely benefit the oral mucosal or periodontal health (Figure 2).

There are some other theoretical bases for the synergistic effects of plaque control with medication on erosive OLP. Firstly, *Candida* spp. may be found on the epithelial surface of OLP patients [36,37]. Yeasts including *Candida albicans* can also be present in periodontal pockets in some periodontitis patients and *Candida* hyphae are reduced after periodontal scaling [38]. *Candida albicans* is a dimorphic yeast associated with tissue invasion and clinical infection that exists as blastoconidia, pseudohyphae, and hypha [39]. Cytokines such as IL-1b, IL-6, and IL-23 can be stimulated by *Candida albicans*, and induced the differentiation of Th17 cells with subsequent generation of IL-17 [40]. As a critical pro-inflammatory cytokine, IL-17 stimulates the expression of tumor necrosis factor-α and IL-6 in keratinocytes, fibroblasts, endothelial cells, and macrophages which may result in OLP erosion [40]. In the buccal mucosa, the epithelial layer is the first barrier that *Candida albicans* has to encounter, oral keratinocyte E-cadherin could be degraded by *Candida albicans* [41]. Virulent hyphae of these *Candida albicans* would cause tissue damage and inflammatory mediator releasement that initiate and sustain local inflammation [40]. Therefore, we speculated that periodontal scaling could decrease the number of *Candida albicans* to accelerate the healing progress of OLP erosion. In addition, Colombo et al. [42] found that the proportion of *Staphylococcus aureus* and *Enterococcus faecalis* colonization on the buccal and gingival membrane was significantly higher in subjects with periodontitis than that in healthy individuals. Polymicrobial biofilms comprising *Candida albicans* and *Staphylococcus aureus* promote oral keratinocyte cell death, apoptosis, and/or necrosis, that increase the frequency and severity of oral diseases [43]. Therefore, it is reasonable to presume that yeasts and bacteria that exist in dental plaque aggravate erosive non-gingival OLP (Figure 2).

The results of the 3-month follow-up showed that the periodontal status (PI and CPI) in the experimental group was better than the control group, indicating excellent oral hygiene maintenance in the experimental group (data not shown). However, there were no significant differences between the two groups in the recurrence rate (Table 4). This demonstrated that poor periodontal condition was not the only basic cause of OLP and was insufficient for erosion initiation. However, it may act as a co-factor with other factors including immune dysfunction and psychological factors to generate erosions.

The intralesional injection caused a minor adverse reaction in only one participant, and that symptom disappeared after 1 h without treatment. We considered that those performing periodontal scaling in OLP patients should be gentle to protect the OLP lesions from a secondary injury. One limitation of this study was that we did not include the reduction of atrophic or white striae as the outcome index. Future studies should add those indices. Due to the scarcity of studies evaluating the effect of periodontal treatment concomitant to medication on the improvement of erosive non-gingival OLP lesions, we point out the importance of conducting further studies of a larger sample that evaluate the long-term adjuvant effect of plaque control. Future studies can also include the erosions that are not close to the gingiva, such as the tongue dorsum and palate.

## 5. Conclusions

This study is the first randomized controlled clinical trial exploring whether plaque control has a short-term synergistic effect with topical corticosteroids on the erosive non-gingival OLP. Although an improvement was observed in patients with plaque control, future studies with larger sample sizes are needed to reinforce the external validity of this study.

## Figures and Tables

**Table 1:** Baseline demographic characteristics.

	Experimental Group (*n* = 25)	Control Group (*n* = 23)	*p* Value
**Age, year (mean ± SD)**	46.5 ± 9.9	49 ± 11.4	0.42
**Sex, number (%)**			0.68
Male	9 (36.0%)	7 (30.4%)	
Female	16 (64.0%)	16 (69.6%)	
**Position, number (%)**			1
Buccal	23 (92.0%)	21 (91.3%)	
Ventral of the tongue	2 (8.0%)	2 (8.7%)	
**OLP duration, month** **(mean ± SD)**	26.2 ± 15.8	31.1 ± 18.9	0.33

The baseline characteristics of the two groups were identical.

**Table 2:** The completely healed percentage of two groups.

Group	Completely Healed (%)	Not Completely Healed (%)	Total
E	23 (95.8%)	1 (4.2%)	24
C	16 (69.6%)	7 (30.4%)	23
Total	39	8	47

The chi-square with Yate’s correction analysis was applied (χ^2^ = 4.03, E vs. C, odds ratio (95% CI) = 10.06 (1.13, 89.94), *p* = 0.045*). E, experimental group; C, control group.

**Table 3:** Comparison of erosion size, NRS score, PI, and CPI between the two groups at the first visit and 14 ± 2 days after the first visit.

	Group	Number	Mean	SD	^b^*p* Value	^c^ Cohen’s d (95% CI)
**Erosion size (mm^2^)**						
First visit	E	25	22.3	20.4	0.40	
	C	23	16.0	12.4		
^a^ Reduced erosion area at day 14 ± 2	E	24	19.1	12.8	0.029 *	0.68 (0.08, 1.28)
	C	23	10.3	13.2		
**NRS score**						
First visit	E	25	4.3	1.8	0.29	
	C	23	4.9	1.7		
^a^ Reduced NRS at day 14 ± 2	E	24	3.9	1.7	0.036 *	0.63 (0.03, 1.23)
	C	23	2.6	2.4		
**PI**						
First visit	E	25	2.6	1.3	0.95	
	C	23	2.5	0.9		
^a^ Reduced PI at day 14 ± 2	E	24	1.7	0.9	<0.001 ***	1.29 (0.65, 1.93)
	C	23	0.6	0.8		
**CPI**						
First visit	E	25	2.0	0.8	0.10	
	C	23	2.4	0.7		
^a^ Reduced CPI at day 14 ± 2	E	24	1.6	0.9	<0.001 ***	1.36 (0.71, 2.01)
	C	23	0.5	0.7		

SD, standard deviation; NRS, numeric rating scale; E, experimental group; C, control group. **^a^** Reduced erosion area/NRS/PI/CPI, was the erosion area/NRS/PI/CPI difference between the first visit and after a 2-week treatment. **^b^ Intergroup Comparison:** Comparison of experimental group and control group by two independent sample *t*-tests. ^c^ Cohen’s d demonstrated the effect size, and it was only calculated for the outcome at day 14 ± 2. *** *p* < 0.001, * *p* < 0.05.

**Table 4:** The recurrence rate of two groups (at 3-month visit) ^a^.

Group	Not Recurred (%)	^b^ Recurred (%)	Total
E	15 (68.2%)	7 (31.8%)	22
C	10 (62.5%)	6 (37.5%)	16
Total	26	12	38

^a^ The chi-square test analysis was applied (*χ^2^* = 0.13, E vs. C, odds ratio (95% CI) = 1.29 (0.33, 4.97), *p* = 0.715). ^b^ the recurred means when a new erosive lesion appeared at the same site as that at the first visit.

## Data Availability

Not applicable.
